# Manual massage versus foam rolling within the NASM corrective framework: a trial for upper crossed syndrome rehabilitation in university students

**DOI:** 10.1038/s41598-026-35030-6

**Published:** 2026-01-27

**Authors:** Mohammad Kalantariyan, Mahmoud Sadeghi, Hadi Samadi

**Affiliations:** Faculty of Sport Sciences, Shahid Rajaee Teacher Training University, Tehran, Islamic Republic of Iran

**Keywords:** Upper crossed syndrome, Manual massage, Foam rolling, Corrective exercise, Postural dysfunction, Health care, Medical research

## Abstract

Upper Crossed Syndrome (UCS) is a prevalent musculoskeletal dysfunction among sedentary young adults, marked by specific patterns of postural misalignment and muscular imbalance. Despite growing reliance on the National Academy of Sports Medicine (NASM) corrective exercise model in conservative management of UCS, a critical gap remains regarding the comparative effectiveness of different myofascial release (MFR) techniques in the foundational “Inhibit” phase. While foam rolling is widely employed for self-MFR, its ability to match the therapeutic depth of manual massage, particularly in accessing deep anterior musculature has yet to be empirically examined in controlled corrective programs. This quasi-experimental, two-arm parallel-group trial evaluated the differential impact of manual massage versus foam rolling within a 12-week NASM-based corrective exercise protocol among 30 male university students (aged 18–25) with diagnosed UCS. Participants were randomized into a Manual Massage Group (MMG) or a Foam Rolling Group (FRG), both performing identical Lengthen–Activate–Integrate phases. Primary outcome variables included postural parameters consisting of Forward Head Angle (FHA), Thoracic Kyphosis Angle (TKA), Rounded Shoulder Angle (RSA), pain intensity, shoulder internal and external range of motion (ROM), upper extremity function, and health-related quality of life. A 2 × 2 mixed-design ANOVA was conducted to evaluate interaction and main effects (*p* < 0.05). Both groups demonstrated significant improvements over time (*p* < 0.001) across all outcome measures. However, the MMG showed significantly greater improvements in RSA (*p* = 0.031), pain reduction (*p* = 0.003), shoulder external and internal ROM (*p* = 0.001 and *p* = 0.016), and SF-36 physical and mental component scores (*p* = 0.001). No significant group-by-time interactions were observed for FHA, TKA, or upper extremity function scores. In the present study, integrating therapist-delivered manual massage was associated with greater improvements in reducing anterior postural deviations, alleviating pain, and enhancing functional mobility and quality of life in UCS populations. These findings support the targeted use of manual MFR in clinical and educational settings where deeper fascial release is warranted. Further research is recommended in diverse populations and long-term follow-ups.

**Supplementary Information:**

The online version contains supplementary material available at 10.1038/s41598-026-35030-6.

## Introduction

Modern sedentary lifestyles marked by prolonged sitting, extensive use of digital devices, and declining levels of physical activity, have contributed significantly to the growing prevalence of musculoskeletal disorders^1^. Among these, Upper Crossed Syndrome (UCS)(Hanif, 2024 #21), first introduced by Janda in 1988, represents a common and clinically important pattern of muscular imbalance^2^.

According to Janda’s model, this pattern arises from a combination of adaptive shortening and facilitation of tonic muscles in the anterior shoulder and posterior cervical region, alongside inhibition and weakness of their phasic antagonists^3,4^. This disrupted synergy alters force couples around the shoulder girdle and cervical spine, leading to the characteristic postural deviations and functional impairments; tight pectorals pull the shoulders forward, increasing Rounded Shoulder Angle (RSA), weak deep neck flexors allow the head to protrude, increasing Forward Head Angle (FHA), and overactive upper trapezius/weak mid-thoracic extensors contribute to increased Thoracic Kyphosis Angle (TKA)^3,5^. The prevalence of UCS is notably high among university students, a population frequently subjected to long hours of sitting and static postures during academic tasks^6,7^. Epidemiological evidence highlights a substantial occurrence; for example, one study reported a 68.3% prevalence of Forward Head Posture (FHP) in this demographic, suggesting an urgent need for early intervention strategies^7^. Beyond aesthetic concerns, UCS has been linked to several clinical complications, including cervicogenic headaches^8^, persistent neck and shoulder pain^9^, restricted cervical and shoulder mobility^10,11^, and potential neurovascular impairments resembling Thoracic Outlet Syndrome (TOS)^12^. Furthermore, increased TKA may adversely affect respiratory mechanics, underscoring the syndrome’s impact beyond the musculoskeletal domain^13^.

Corrective exercise (CEx) protocols have become a cornerstone in non-pharmacological management of UCS, offering a structured, low-risk, and cost-effective approach to restoring postural and functional balance^10,14^. The widely adopted National Academy of Sports Medicine (NASM) Corrective Exercise Specialist (CES) model consists of a four-phase progression: Inhibit, Lengthen, Activate, and Integrate^4,14,15^. The “Inhibit” phase is designed to reduce excessive muscle tension through myofascial release (MFR), thereby preparing tissues for subsequent flexibility and strengthening interventions^15^. The rationale for incorporating targeted strengthening (Activate and Integrate phases) is to address the inhibited weak muscles identified in the UCS pattern. For instance, strengthening the mid-thoracic extensors, lower trapezius and serratus anterior is crucial for restoring scapular upward rotation and posterior tilting, which directly counteracts rounded shoulders and facilitates improved glenohumeral kinematics^3,33^. Research demonstrates that such targeted activation, following inhibition and lengthening, leads to more sustainable postural correction than stretching alone^10,32^.

Foam rolling, a self-administered form of MFR, is commonly used during the Inhibit phase due to its practicality and accessibility^15^. Its proposed mechanisms include mechanical stimulation of mechanoreceptors, autonomic nervous system modulation, and improved fascial mobility through alterations in viscoelastic properties^16,17^. Despite its benefits, foam rolling has notable limitations, such as difficulty targeting deeper muscle groups such as pectoralis minor, inconsistent pressure control, and user discomfort, particularly among inexperienced individuals which may affect adherence and treatment outcomes^18^.

Manual massage, on the other hand, is a therapist-applied MFR technique that allows for more precise tissue engagement, customizable pressure, and potentially greater patient relaxation^17^. Its physiological effects include enhanced circulation, modulation of pain perception, reduced muscle reflex excitability, and activation of the parasympathetic nervous system^19,20^. Clinical studies support its efficacy in reducing musculoskeletal pain, increasing range of motion (ROM), and improving overall muscular function across varied populations^21^.

Although both foam rolling and manual massage are recognized components of MFR within the NASM framework, there remains a significant gap in the literature regarding their comparative effectiveness during the Inhibit phase, especially in university students with UCS. While some studies have incorporated self-myofascial release into NASM-based interventions^15,22^, direct comparisons between foam rolling and manual massage are lacking. Given the foundational role of the Inhibit phase in facilitating overall program success, determining whether the choice of MFR technique significantly influences clinical outcomes is of practical and therapeutic importance. Therefore, this study aimed to determine whether the type of MFR modality employed in the Inhibit phase influences clinical and functional outcomes in young adults with UCS.

## Methodology

### Study design

**Fig. 1:**
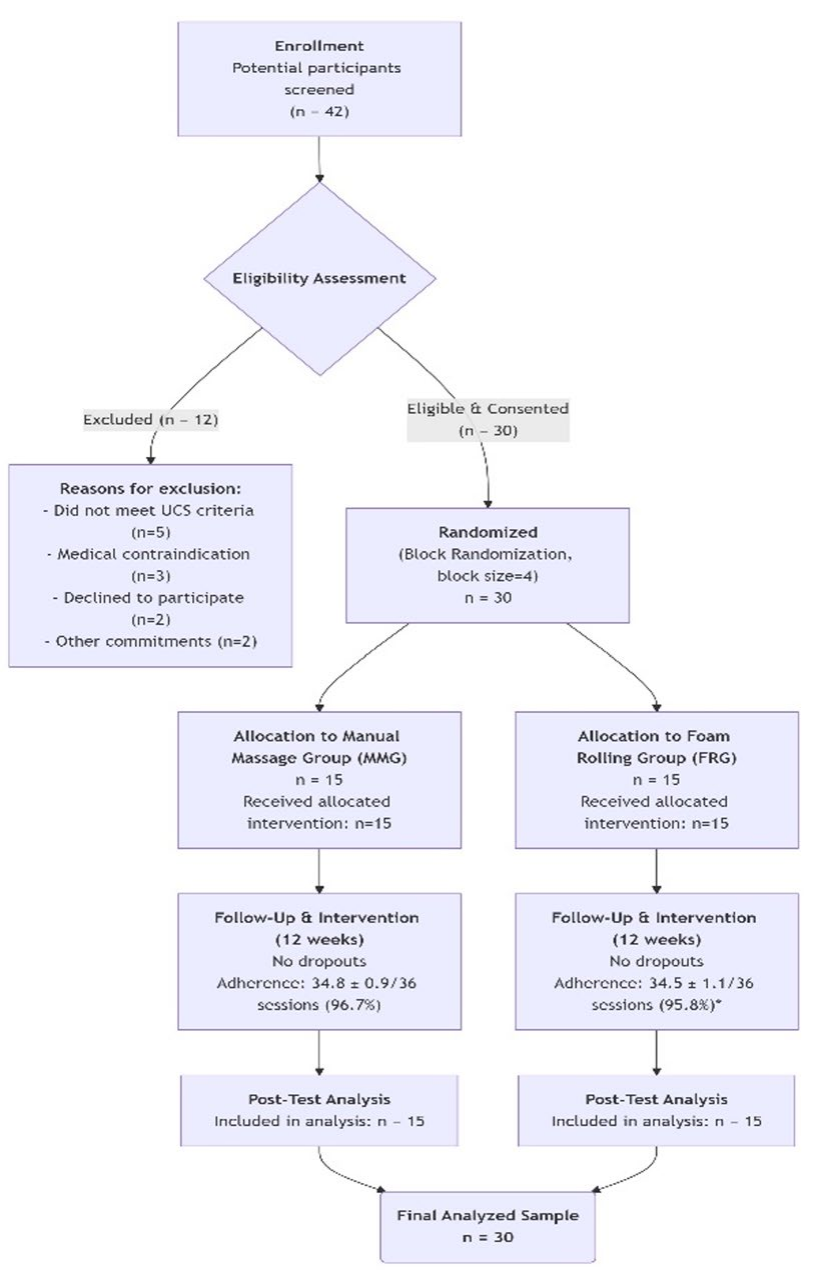
CONSORT flow diagram of participant enrollment, allocation, follow-up, and analysis.

This study employed a parallel-group, assessor-blinded, quasi-experimental design with pre- and post-intervention assessments. Participants were allocated to one of two intervention arms using a computer-generated block randomization sequence (block size = 4) to ensure group balance at baseline. The study is classified as quasi-experimental because, while allocation was randomized, full blinding of participants and therapists to the intervention was not feasible, and the study did not include a passive control group (no treatment or placebo). The study design and participant flow are summarized in Fig. 1.

### Ethics approval

The study protocol and all study procedures were reviewed and approved by the Ethics Committee of Shahid Rajaee Teacher Training University. All procedures were performed in accordance with relevant institutional guidelines and regulations and with the principles of the Declaration of Helsinki. Prior to participation, each participant received a full verbal and written explanation of the study and provided written informed consent. Participant confidentiality was safeguarded by removing direct identifiers and assigning unique study codes; study data were stored on encrypted institutional servers with access restricted to authorized study personnel only. Although no formal approval reference number is available in our records, the Ethics Committee reviewed and approved the conduct of this study. Written confirmation of approval from the Committee Chairman, dated November- 2024, is available upon request.

### Participants and recruitment

Participants were recruited from male undergraduate engineering students through formal announcements circulated via university communication platforms. Eligibility screening was conducted by trained research staff based on pre-established criteria. A priori sample size estimation using G*Power (version 3.1.9.7) indicated that 14 participants per group were needed to detect a large effect size (Cohen’s f = 0.40; equivalent to d = 0.80) with 85% power at a 0.05 significance level in a mixed-design ANOVA. Accounting for potential attrition, a total of 30 eligible participants were enrolled. The sample size estimation was based on detecting a large effect (Cohen’s f = 0.40), as preliminary studies and meta-analyses on corrective exercise for UCS often report large within-group improvements in postural and pain outcomes^5,10^. Furthermore, we aimed to be adequately powered to detect a potentially meaningful difference between two active interventions.

Inclusion criteria were: male undergraduate engineering students aged 18–25 years; completion of at least six academic semesters; reported computer usage ≥ 35 h per week; low physical activity (≤ 2 structured exercise sessions per week over the past six months); and postural deviations consistent with UCS, operationally defined as: (a) TKA > 45° (exceeding the upper limit of the normative range of 20° to 45° for young adults), (b) FHA > 48°, and (c) RSA > 52°^10,22^. Participants also needed to report neck and/or shoulder pain with a minimum VAS score of 3.5/10^23^.

Exclusion criteria included diagnosed spinal conditions (e.g., scoliosis, disc herniation, spondylolisthesis) requiring medical intervention^22^, neurological disorders affecting the upper quadrant^13^, cervical osteoarthritis^22^, recent trauma or surgery (< 6 months), contraindications to massage or foam rolling (e.g., dermatological or inflammatory conditions)^19^, or an inability/unwillingness to adhere to the full 12-week protocol. After eligibility confirmation and consent, participants completed a health history questionnaire and were randomized into either the MMG or FRG (n = 15 per group) using a computer-generated block randomization method (block size = 4) to ensure group balance.

### Outcome measures

All assessments were performed at baseline and following the 12-week intervention. To confirm measurement consistency within our lab and sample, intra-rater reliability was assessed prior to the main trial. The primary assessor performed all measurements (FHA, RSA, TKA) on 10 pilot subjects (with UCS characteristics) on two separate days, 48 h apart. The Intraclass Correlation Coefficient (ICC) for single measures demonstrated excellent reliability: FHA (ICC = 0.93, 95% CI: 0.79–0.98), RSA (ICC = 0.95, 95% CI: 0.84–0.99), and TKA (ICC = 0.91, 95% CI: 0.72–0.97). Anthropometrics: Height and body mass were recorded using a calibrated stadiometer and digital scale (Seca GmbH & Co. KG, Hamburg, Germany).

### Postural assessment

**Fig. 2:**
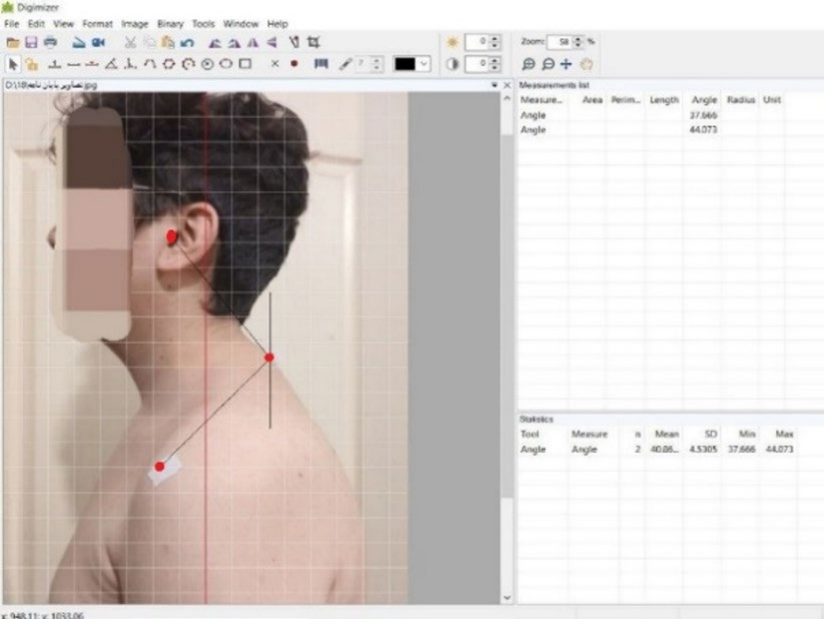
Photogrammetric setup for assessing FHA and RSA. Anatomical landmarks (tragus, acromion, C7) are indicated.

FHA and RSA were measured using standardized 2D sagittal photogrammetry^10^. Participants stood in a relaxed stance with feet shoulder-width apart, looking straight ahead at a wall-mounted marker at eye level. Markers were placed on the skin overlying the following anatomical landmarks by the same assessor: tragus of the ear, acromion process (posterior-lateral edge), and spinous process of the 7th cervical vertebra (C7). Lateral images were captured using a Canon EOS 80D digital camera positioned at a fixed distance and height. Image analysis was conducted with Digimizer software (v5.4.9) (Fig. 2). The average of three image-based measurements was used. This method has good reproducibility, such that Ruivo et al., reported intra- and inter-examiner reliability for FHA and for RSA^24^. TKA was measured using a calibrated dual digital inclinometer. Inclinometer was placed over palpated landmarks (T2/T3 and T11/T12) in a relaxed standing posture. The average of three readings was recorded. The manual inclinometer is recommended as a valid instrument for measuring TKA, with good agreement with the gold standard. Postural angle changes of ≥ 5 degrees in FHA, TKA, or RSA are generally regarded as clinically significant in corrective exercise interventions targeting UCS^25^.

### Pain intensity

Neck/shoulder pain during the previous week was measured via a 100-mm horizontal Visual Analog Scale (VAS)^22^. The left end of the VAS represented ‘no pain’ and the right end shows ‘most severe pain imaginable’. The VAS is simple and efficient to use and has been shown to be reliable and valid as a ratio-scale measure of pain intensity. A change of ≥ 1.5 cm on the VAS is generally considered the Minimal Clinically Important Difference (MCID) for musculoskeletal pain, indicating a perceptible and meaningful improvement in symptoms^26^.

### Range of motion (ROM)

Shoulder external and internal rotation ROM were assessed using a Digital Inclinometer (model ACU360; Lafayette Instrument Co, Lafayette, IN, USA). The manufacturer’s specifications indicate that this instrument is capable of measuring a range up to 180 with an accuracy of 1 degree. For the ROM measurements with the inclinometer, the digital inclinometer was zeroed by using a fixed horizontal or vertical reference point, depending on the measurement, to ensure accuracy. For inclinometric measurements, scapular compensation was controlled by palpating the coracoid (with the examiner’s thumb) and the spine of the scapula (with the examiner’s fingers). This method has been shown to more accurately control for scapular movements compared with putting the full hand on the shoulder during the measurement. Three familiarization trials were performed for each measurement, and 3 subsequent trials were executed for further analysis. Reported reliability for these measures ranges from ICC 0.89–0.99. Improvements of ≥ 10 degrees in shoulder ROM are regarded as clinically meaningful based on MCID thresholds reported in upper extremity rehabilitation studies^27^.

### Upper extremity function

The Upper Quarter Y-Balance Test (UQYBT) was used to assess functional performance^28^. To perform this test, the individual stood on their hands and toes, keeping their spine and lower limbs in a straight line. The dominant hand was chosen as the support. The position of the hand was marked by a designated line, and the feet were separated by the width of the shoulders. In this position, the individual was asked to reach as far as possible in the medial, inferior-lateral, and superior-lateral directions with their free hand while maintaining the position of the supporting hand, torso, and lower limbs. To enable comparisons between individuals, the reach distances along the upper limb (the distance from the seventh cervical vertebra to the end of the longest finger in 90 degrees of shoulder abduction and elbow extension, wrist and finger extension) were normalized. The reaching task was performed continuously in all three directions without rest, and the individual was allowed to rest their free hand on the ground after each trial. This process was repeated three times, and the average of the three correct trials in all three directions was recorded as the composite score of the individual in the functional stability test. The intra-tester reliability of this test was reported as good to excellent (0.80–0.90). Although a standardized MCID for UQYBT is not firmly established, improvements ≥ 8–10% in composite scores are commonly interpreted as functionally relevant in upper limb performance assessment^29^.

### Health-related quality of life (HRQoL)

To assess HRQoL, SF-36 was used. The SF-36 is a general health related quality of life survey which comprises 36 multiple choice questions sorted in eight categories or subscales which address health constructs considered to be important to most health care situations and generally covers two basic domains including physical health and mental health. These items are as follows physical functioning, role limitations (physical problems), bodily pain, general health, vitality, social functioning, role limitations (emotional problems), and mental health. A 5-point change in SF-36 component scores is typically considered the MCID, reflecting a meaningful enhancement in perceived physical or mental health status^26^. The questionnaire is translated to Farsi and validated in Iran with the reliability and validity of (a = 0.76, validity > 0.8)^30^.

### Intervention protocols

The 12-week intervention was structured according to the NASM Corrective Exercise Continuum (CEC), comprising four distinct yet overlapping phases: Inhibit, Lengthen, Activate, and Integrate (Table 1). It is important to clarify that these phases are not mutually exclusive but are designed to be progressively emphasized. For instance, while the primary focus of Weeks 1–4 is Inhibition, elements of this phase continue at a reduced volume (1 set vs. 1–3 sets initially) into Weeks 5–8 to maintain tissue compliance as the emphasis shifts to Lengthening. This overlapping design is a core feature of the NASM model, ensuring that gains in one phase are supported and maintained throughout the subsequent phases^4^^,^^15^. Each session (lasting approximately 45–60 min) was led by certified CES and structured according to the four phases of the NASM Corrective Exercise model^4^. The only difference between groups lay in the modality used during the Inhibit phase (See Table 1). All 36 subsequent exercise sessions were fully supervised by NASM CES, who provided immediate verbal and tactile feedback to correct form in real-time.

**Table 1:** NASM-based corrective exercise protocol for upper cross syndrome.

Target muscle		Sets	Duration	Notes	
Phase 1: inhibition (weeks 1–4)					
Latissimus dorsi		1–3	30 sec	Thera cane & foam roll	
Thoracic spine		1–3	30 sec	Thera cane	
Upper trapezius		1–3	30 sec	Thera cane	
Sternocleidomastoid		1–3	30 sec	Thera cane	
Levator scapula		1–3	30 sec	Thera cane	

Target muscle		Sets	Duration	Notes	
Phase 2: inhibition (weeks 5–8)					
Latissimus dorsi		1	30 sec	Thera cane & foam roll	
Thoracic spine		1	30 sec	Thera cane	
Upper trapezius		1	30 sec	Thera cane	
Sternocleidomastoid		1	30 sec	Thera cane	
Levator scapula		1	30 sec	Thera cane	

Stretch exercise		Sets	Duration	Notes	
Phase 2: lengthening (weeks 5–8)					
Sternocleidomastoid stretch		1–3	30 sec	Stretching	
Levator scapulae stretch		1–3	30 sec	Stretching	
Upper trapezius stretch		1–3	30 sec	Stretching	
Ball latissimus dorsi stretch		1–3	30 sec	Using ball	
Standing pectoral stretch		1–3	30 sec	Stretching	

Stretch exercise		Sets	Duration	Notes	
Phase 3: lengthening (weeks 9–12)					
Sternocleidomastoid stretch		1–2	30 sec	Stretching	
Levator scapulae stretch		1–2	30 sec	Stretching	
Upper trapezius stretch		1–2	30 sec	Stretching	
Ball latissimus dorsi stretch		1–2	30 sec	Using ball	
Standing pectoral stretch		1–2	30 sec	Stretching	

Exercise	Sets	Reps	Tempo	Rest	Target muscle group
Phase 4: isolated activation (weeks 9–12)					
Quadruped ball chin tucks *	1–2	10–15	4/2/2	0	Deep cervical flexors
Resisted cervical posterior translation *	1–2	10–15	4/2/2	0	Cervical-thoracic extensors
Floor prone scaption *	1–2	10–15	4/2/2	0	Lower trapezius
Ball combo I	1–2	10–15	4/2/2	0	Isolated activation
*: First three exercises are repeated for reinforcement.					

Exercise	Sets	Reps	Tempo	Rest	Notes
Phase 4: integrated dynamic movement (weeks 9–12)					
Ball combo I with cervical retraction	1–2	10–15	Slow	30 sec	Using ball
Squat to row	1–2	10–15	Slow	30 sec	Functional integration
Single-leg romanian deadlift	1–2	10–15	Slow	30 sec	Functional integration
Standing one-arm cable chest press	1–2	10–15	Slow	30 sec	Functional integration

*Phase 1*: Inhibition (Weeks 1–4)—Primary Emphasis. For both Manual Massage Group (MMG) and Foam Rolling Group (FRG), applied pressure was standardized to a subjectively reported ‘optimal release pressure’ corresponding to 5–7 on a 10-point Numeric Rating Scale (NRS), where ‘1’ is no sensation and ‘10’ is intolerable pain. This scale was verbally anchored for participants: ‘A feeling of strong, therapeutic pressure that may be uncomfortable but not painful.’ Participants were instructed to provide verbal feedback during every session (e.g., ‘reduce pressure,’ ‘good pressure’), and therapists adjusted accordingly. This method is clinically validated for eliciting myofascial release without triggering protective muscle guarding^31,37^.

*MMG*: The manual massage was administered by a single certified NASM CES holding a Master’s degree in Corrective Exercise (CE) with over 5 years of hands-on clinical experience to ensure consistency. Techniques included superficial/deep effleurage (~ 2 min per region), petrissage (~ 3 min per muscle), and ischemic compression (30–60 s) on trigger points at 5–7/10 subjective discomfort levels^31^.

*FRG*: FRG participants underwent a two-part, competency-based training protocol before commencing the intervention: (a) a 60-min group workshop covering theory and demonstration, and (b) an individual 30-min practical session where each participant demonstrated correct technique for all target muscles under supervision. Competency was defined as the ability to independently perform the roll with controlled speed (~ 2–3 cm/sec), sustain pressure on a tender point for 20–30 s without breath-holding, and identify improper bony contact.

Following the initial Inhibit phase, the protocol progressed through the subsequent three phases of the NASM model: Lengthen (Weeks 5–8), Activate (Weeks 9–12), and Integrate (Weeks 9–12). These phases were implemented in a progressive, overlapping manner for both intervention groups, with all exercises performed under the direct, real-time supervision of a CES to ensure strict adherence to technique and safety. The specific FITT (Frequency, Intensity, Time, Type) principles and progression criteria for each phase are detailed below.

*Phase 2*: Lengthen (Weeks 5–8)—Primary Emphasis. The objective of this phase was to improve soft-tissue extensibility and restore optimal muscle length. Participants performed static stretches targeting the pectoralis major/minor, upper trapezius, levator scapulae, sternocleidomastoid, and latissimus dorsi. Each stretch was held for 30 s. The stretch intensity was subjectively monitored to maintain a perceptible but non-painful stretch (≤ 7/10 on a numeric scale). Number of sets progressed from 1–3 sets per muscle in weeks 5–8 to 1–2 sets in the latter 4 weeks. A maintenance dose of the assigned Inhibit technique (1 set of 30 s per muscle) continued throughout this phase to sustain tissue compliance.

*Phase 3*: Activate (Weeks 9–12)—Primary Emphasis. This phase aimed to re-educate and strengthen the inhibited, phasic muscles identified in the UCS pattern. Exercises included isolated, low-load movements such as quadruped chin tucks, resisted cervical retraction, and prone scaption. Overload parameters were based on bodyweight or minimal external resistance with a focus on precise movement control. Number of sets involved 1–2 sets of 10–15 repetitions with a controlled 4/2/2 (eccentric/isometric/concentric) tempo. Progression from 1 to 2 sets was permitted only after the supervising CES confirmed the participant could execute all repetitions with perfect form and without compensatory movements for two consecutive sessions.

*Phase 4*: Integrate (Weeks 9–12)—Concurrent Emphasis. The final phase focused on stabilizing corrections through dynamic, multi-joint, and functional movement patterns. Exercises included integrated drills such as squat-to-row and single-leg Romanian deadlift. Intensity remained low to moderate with primary focus on movement quality. Number of sets involved 1–2 sets of 10–15 repetitions performed with a controlled tempo and 30-s rest intervals. Progression criteria mirrored those of the Activate phase, requiring mastery of technique and control.

Fidelity to the protocol was rigorously monitored. For the MMG, therapists completed a per-session checklist for each participant, documenting the muscles addressed, techniques used, and the participant’s reported NRS pressure level. For the FRG, the supervising specialist completed a similar technique quality log per session per participant, rating key components (posture, speed, pressure sustainment) on a 3-point scale (correct/needed minor cueing/incorrect). Random audits of 20% of sessions were performed by the principal investigator. Fidelity was maintained at > 95% for both groups, with no major deviations. All 30 enrolled participants completed the full 12-week protocol and post-test assessments. Adherence to the supervised intervention sessions was meticulously tracked and was exceptionally high in both groups. All 30 enrolled participants (100%) completed the full 12-week intervention and the post-test assessment, resulting in a 0% attrition rate. The mean attendance, calculated as the number of sessions attended out of the 36 scheduled sessions, was 34.8 ± 0.9 sessions in the MMG (96.7% adherence rate) and 34.5 ± 1.1 sessions in the FRG (95.8% adherence rate). No participants were excluded from the final analysis due to non-adherence or dropout.

### Statistical analysis

All data were analyzed using IBM SPSS Statistics for Windows, Version 26.0 (IBM Corp., Armonk, NY). All dependent variables met the assumptions of normality (Shapiro–Wilk test, all *p* > 0.05) and homogeneity of variance (Levene’s test, all *p* > 0.05). Given the exploratory nature of comparing two active treatments across multiple related but distinct domains of UCS (posture, pain, function, HRQoL), and to avoid an over-conservative increase in Type II error, we did not apply an alpha correction for multiple comparisons for the primary interaction effects. This approach is consistent with similar comparative efficacy trials in rehabilitation research^31^. Our interpretation prioritizes effect sizes and clinical significance (MCID) alongside *p*-values. Independent t-tests were used to compare baseline group characteristics. A 2 (Group: MMG vs. FRG) × 2 (Time: Pre vs. Post) mixed-design ANOVA was performed for each outcome. Partial eta-squared (ηp^2^) was reported as the effect size. Statistical significance was defined as *p* < 0.05.

## Results

Baseline demographic characteristics are presented in Table 2. Independent samples t-tests revealed no statistically significant differences between the MMG and FRG at baseline for age, height, weight, or body mass index (BMI) (all *p* > 0.05).

**Table 2:** Baseline demographic characteristics of participants by group.

Variable	MMG Mean ± SD	FRG Mean ± SD	*p*-value
Age (years)	21.5 ± 1.8	21.9 ± 2.1	0.323
Height (cm)	178.3 ± 8.2	180.1 ± 6.9	0.437
Weight (kg)	81.7 ± 4.7	83.2 ± 5.1	0.255
BMI (kg.m^2^)	24.4 ± 3.1	23.6 ± 2.5	0.359

MMG, manual massage group; FRG, foam rolling group; BMI, body mass index.

Pre- and post-intervention means and standard deviations for all outcome measures are presented in Table 3. The results of the 2 (Group: MMG, FRG) × 2 (Time: Pre, Post) mixed-design ANOVAs are summarized in Table 4.

**Table 3:** Pre- and post-intervention scores (Mean ± SD) for all outcome measures by group.

Dependent variable	Group	Pre-test Mean ± SD	Post-test Mean ± SD
TKA (deg)	MMG	47.8 ± 2.9	40.5 ± 3.6
	FRG	48.1 ± 3.3	40.1 ± 3.2
FHA (deg)	MMG	52.1 ± 4.5	44.5 ± 3.8
	FRG	51.8 ± 4.2	44.8 ± 4.1
RSA (deg)	MMG	55.6 ± 5.1	47.5 ± 4.0
	FRG	56.0 ± 4.8	50.1 ± 4.5
VAS Score (0–10)	MMG	5.3 ± 1.1	2.8 ± 0.8
	FRG	5.1 ± 1.3	3.5 ± 1.0
Shoulder EX ROM (deg)	MMG	53.5 ± 6.2	64.3 ± 5.5
	FRG	55.2 ± 5.9	61.9 ± 5.1
Shoulder IR ROM (deg)	MMG	45.8 ± 4.0	56.2 ± 4.9
	FRG	46.5 ± 4.5	53.0 ± 5.1
UQYBT Composite (%)	MMG	85.4 ± 5.5	93.1 ± 4.8
	FRG	86.0 ± 6.1	92.5 ± 5.2
SF-36 PCS	MMG	40.1 ± 6.3	52.5 ± 5.5
	FRG	41.5 ± 6.8	48.8 ± 6.0
SF-36 MCS	MMG	45.8 ± 7.0	53.9 ± 6.2
	FRG	46.5 ± 6.5	51.1 ± 5.8

TKA, thoracic kyphosis angle; FHA, forward head angle; RSA, rounded shoulder angle; VAS, visual analog scale; ER, external rotation; IR, internal rotation; ROM, range of motion; UQYBT, upper quarter Y-balance test; PCS, physical component summary; MCS, mental component summary.

**Table 4:** Summary of mixed-design ANOVA results (Group × Time) for all outcome measures.

Dependent variable	Effect	F (1, 28)	*p*-value	Partial η^2^
FHA (deg)	Interaction	3.51	0.066	0.060
	Time	45.82	**0.001**	0.454
	Group	0.89	0.350	0.016
RSA (deg)	Interaction	4.88	**0.031**	0.081
	Time	52.15	**0.001**	0.486
	Group	1.21	0.276	0.022
TKA (deg)	Interaction	0.47	0.229	0.104
	Time	59.21	**0.001**	0.612
	Group	0.91	0.417	0.035
VAS score (0–10)	Interaction	9.65	**0.003**	0.149
	Time	110.2	**0.001**	0.667
	Group	3.15	0.081	0.054
Shoulder EX ROM (deg)	Interaction	13.11	**0.001**	0.037
	Time	35.70	**0.001**	0.593
	Group	0.50	0.481	0.009
Shoulder IR ROM (deg)	Interaction	6.23	**0.016**	0.102
	Time	68.91	**0.001**	0.556
	Group	2.55	0.116	0.044
UQYBT Composite score (%)	Interaction	0.75	0.390	0.013
	Time	75.33	**0.001**	0.578
	Group	0.12	0.730	0.002
SF-36 PCS	Interaction	9.89	**0.001**	0.033
	Time	41.12	**0.001**	0.628
	Group	0.66	0.421	0.012
SF-36 MCS	Interaction	11.45	**0.001**	0.008
	Time	25.50	**0.001**	0.517
	Group	0.09	0.765	0.002

Effect: Interaction = Group × Time; Time = Main effect of Time; Group = Main effect of Group. Bold *p*-values indicate statistical significance (p < 0.05). Partial η^2^ benchmarks: ≥ 0.01 small, ≥ 0.06 medium, ≥ 0.14 large.

Detailed within-group change scores, their 95% confidence intervals, and the proportion of participants achieving the MCID are presented in Supplementary Table S1.

Significant main effects of Time were observed for all dependent variables (all *p* < 0.001), indicating substantial overall improvements from pre-test to post-test across both groups combined.

Significant Group × Time interaction effects were found for RSA, VAS pain score, shoulder ER ROM, shoulder IR ROM, SF-36 PCS, and SF-36 MCS (all p < 0.05, Table 4). Inspection of the means in Table 3 indicates that the MMG demonstrated greater pre-to-post improvement than the FRG for these specific variables. However, the magnitude of these between-group differences, as indicated by partial eta-squared (η^2^), varied considerably. According to conventional benchmarks, the interaction effect sizes ranged from trivial (e.g., SF-36 MCS, η^2^ = 0.008) to large (e.g., VAS, η^2^ = 0.149).

No significant Group × Time interactions were observed for FHA, TKA, or the UQYBT composite score (all p > 0.05), suggesting that the magnitude of improvement for these outcomes did not differ significantly between the MMG and FRG. Furthermore, no significant main effects of Group were found for any variable (all p > 0.05).

## Discussion

This study sought to evaluate the comparative efficacy of integrating either manual massage or foam rolling into the NASM corrective exercise continuum for managing UCS in university students. The notable improvements observed across all outcome variables in both groups affirm the effectiveness of the NASM framework itself. These findings are consistent with existing literature emphasizing the value of comprehensive corrective exercise programs in restoring musculoskeletal symmetry in UCS populations^4,10,32^.

Direct comparisons of manual massage and foam rolling within a structured CEx protocol are scarce. However, our findings align with the study by Lukasić et al.^31^, which also reported superior benefits of manual myofascial techniques over standard massage for pain and posture in UCS. Conversely, a review by Wilke et al.^37^ concluded that foam rolling primarily affects ROM with limited impact on static posture, which is consistent with our findings of non-significant between-group differences for FHA and TKA. This underscores the notion that the choice of MFR technique may be outcome-specific.

Crucially, while both interventions produced clinically meaningful improvements, integrating therapist-delivered manual massage was associated with comparative improvements in several domains. Specifically, the data suggest a comparative advantage for manual massage in improving RSA, pain reduction, shoulder internal and external rotation ROM, and both physical and mental aspects of HRQoL. These results suggest that the specificity and depth afforded by manual massage may offer additional therapeutic benefit when addressing anterior shoulder tightness and associated dysfunction.

In contrast, variables such as FHA, TKA, and UQYBT performance improved similarly in both groups. This implies that while MFR is essential as a preparatory intervention, the subsequent activation and integration phases of the NASM model likely exert a more dominant influence on these neuromuscular and postural outcomes. For instance, improvements in FHA and TKA are heavily dependent on re-educating deep cervical flexors and thoracic extensors that targets addressed more thoroughly in the later corrective phases^4^. Similarly, UQYBT performance reflects dynamic motor control and scapulohumeral stability, which are refined during activation and integration rather than initial tissue release^33^.

The observed greater RSA correction in the MMG could be explained by the therapist’s ability to apply more focused pressure to the pectoralis minor and other anterior fascial restrictions; the structures that are often difficult to access effectively using a foam roller^31,34^. Improved scapular mechanics resulting from better anterior chest wall extensibility likely contributed to the observed gains in shoulder mobility, particularly in internal and external rotation^35^. These biomechanical adjustments not only improve functional range but also reduce strain on the posterior kinetic chain, potentially explaining the MMG group’s superior pain reduction^19^. These proposed mechanisms while plausible and grounded in existing physiological literature, remain hypothetical within the constraints of the present study design, which lacked direct measures of fascial stiffness or neurophysiological activity. Future research incorporating tools such as ultrasound elastography or quantitative sensory testing is needed to validate these pathways.

Moreover, the analgesic advantage of manual massage may derive from both mechanical and neurophysiological mechanisms^19,21^. Direct manual pressure can more effectively deactivate myofascial trigger points^20^, while also activating segmental inhibitory pathways such as gate control mechanisms and descending pain modulation^19,36^. Additionally, the psychosocial aspects of receiving hands-on care such as increased relaxation and perceived therapeutic attention, may further influence pain perception^36^ and contribute to improved adherence and exercise quality throughout the program^19^.

The marked improvements in SF-36 physical and mental component scores among MMG participants likely reflect a synergistic benefit: enhanced posture, pain relief, restored function, and a sense of well-being stemming from direct human interaction^36^. While foam rolling is recognized for its practicality and effectiveness, particularly in enhancing tissue hydration and viscoelasticity^16^, it may lack the adaptability and targeted precision required to elicit comparable improvements in structural impairments such as RSA. Our findings support previous research indicating that foam rolling alone has limited impact on static postural outcomes specially in male people^18,37^.

Taken together, these results suggest that while both MFR techniques are valuable tools within a corrective exercise program, manual massage may be the more effective choice when treating UCS cases where anterior structural restrictions, shoulder ROM deficits, and pain are primary concerns. In contrast, for outcomes driven primarily by neuromuscular control and postural retraining, both modalities appear equally sufficient as precursors to the activation and integration phase.

### Strengths and limitations

This study presents several notable strengths. It is among the few to directly compare manual massage and foam rolling within a structured and clinically relevant corrective exercise framework. By evaluating a broad spectrum of outcomes ranging from objective posture and function to subjective pain and quality of life, the research provides a holistic view of therapeutic effectiveness. The focus on university students also addresses a high-risk yet often overlooked population.

This study has some limitations. The datasets include sensitive participant information and cannot be made publicly available; however, de-identified data and analysis code can be shared upon reasonable request, subject to Ethics Committee approval. The sample was exclusively male. This choice was made primarily for internal homogeneity regarding risk factor exposure (prolonged sitting in engineering curricula) in this initial comparison. Furthermore, in the context of this study, specific cultural and ethical guidelines posed significant challenges for the recruitment and physical assessment of female participants for a trial involving manual therapy and postural evaluation. While this limits the generalizability of our findings to females and other populations, it was a necessary constraint within the present research context. Future studies across different cultural settings with mixed-gender cohorts are essential. In addition, the absence of a follow-up assessment after the completion of the 12-week program means we cannot determine the durability of the observed improvements. Future studies should replicate these findings in larger and more diverse cohorts, incorporate longitudinal designs, and use objective mechanistic measures (e.g., elastography, surface EMG, biomarkers) or comparative trials with other myofascial release strategies to clarify underlying mechanisms and relative effectiveness. We also acknowledge the blinding limitation; While assessors were blinded, the nature of the interventions prevented blinding of participants and therapists, which may have introduced performance bias. We attempted to mitigate this by using objective outcome measures where possible and standardizing all other aspects of the protocol.

## Conclusion

This study confirms that both therapist-administered manual massage and supervised foam rolling, when incorporated into a NASM-based CEs protocol, can significantly enhance posture, reduce musculoskeletal pain, increase shoulder ROM, improve upper extremity function, and elevate HRQoL in university students with UCS. However, the data suggest a comparative advantage for manual massage in improving RSA, reducing pain, increasing shoulder mobility, and enhancing both physical and mental quality of life indices. While foam rolling remains an accessible and effective method of MFR, manual massage may be the preferred approach in clinical settings where the goal is to target deeper fascial restrictions and maximize structural correction. Ultimately, the choice of MFR technique should be guided by clinical presentation, therapeutic goals, available resources, and patient preference, always within the context of a structured, phase-based corrective exercise plan.

## Supplementary Information

Below is the link to the electronic supplementary material.

Supplementary Material 1

## Data Availability

The datasets generated and/or analysed during the current study are not publicly available due to participant confidentiality restrictions. De-identified datasets that support the findings of this study are available from the corresponding author, upon reasonable request and subject to approval by the Ethics Committee of Shahid Rajaee Teacher Training University and to the execution of an appropriate data-sharing agreement. Detailed methodological descriptions and aggregated summary statistics are provided in the manuscript and Supplementary Information to facilitate reproducibility.

## Abbreviations

UCS: Upper crossed syndrome
NASM: National academy of sports medicine
CES: Corrective exercise specialist
MFR: Myofascial release
MMG: Manual massage group
FRG: Foam rolling group
ROM: Range of motion
TKA: Thoracic kyphosis angle
FHA: Forward head angle
RSA: Rounded shoulder angle
VAS: Visual analog scale
SF-36: 36-Item short form health survey
PCS: Physical component summary
MCS: Mental component summary
UQYBT: Upper quarter Y-balance test
ANOVA: Analysis of variance
ICC: Intraclass correlation coefficient
MCID: Minimal clinically important difference
