# Supplementary material for: Manual massage versus foam rolling within the NASM corrective framework: a trial for upper crossed syndrome rehabilitation in university students
Source: Sci Rep. 2026 Jan 27;16:5471. doi: 10.1038/s41598-026-35030-6 (PMC12886921; doi:10.1038/s41598-026-35030-6)
Supplement: Supplementary file 1 — Supplementary Material 1 [file 41598_2026_35030_MOESM1_ESM.pdf]

Supplementary Table S1. Within-group changes from baseline to post-intervention, with 95% confidence intervals and the proportion of participants achieving the Minimal Clinically Important Difference (MCID).

Dependent Variable	Group	Pre-test Mean ± SD	Post-test Mean ± SD	Mean Change (Post-Pre)	95% CI for Mean Change	MCID Threshold	Participants Achieving MCID, n (%)
TKA (deg)	MMG	47.8 ± 2.9	40.5 ± 3.6	-7.60	(-9.74, -5.46)	≥5.0°	13 (86.7%)
	FRG	48.1 ± 3.3	40.1 ± 3.2	-7.00	(-8.99, -5.01)		12 (80.0%)
FHA (deg)	MMG	52.1 ± 4.5	44.5 ± 3.8	-8.10	(-10.36, -5.84)	≥5.0°	14 (93.3%)
	FRG	51.8 ± 4.2	44.8 ± 4.1	-5.90	(-7.88, -3.92)		11 (73.3%)
RSA (deg)	MMG	55.6 ± 5.1	47.5 ± 4.0	-7.30	(-9.08, -5.52)	≥5.0°	14 (93.3%)
	FRG	56.0 ± 4.8	50.1 ± 4.5	-8.00	(-9.70, -6.30)		14 (93.3%)
VAS Score (0-10)	MMG	5.3 ± 1.1	2.8 ± 0.8	-2.50	(-3.01, -1.99)	≥1.5 cm	15 (100%)
	FRG	5.1 ± 1.3	3.5 ± 1.0	-1.60	(-2.18, -1.02)		12 (80.0%)
Shoulder EX ROM (deg)	MMG	53.5 ± 6.2	64.3 ± 5.5	+10.80	(+8.26, +13.34)	≥10.0°	12 (80.0%)
	FRG	55.2 ± 5.9	61.9 ± 5.1	+6.70	(+4.52, +8.88)		8 (53.3%)
Shoulder IR ROM (deg)	MMG	45.8 ± 4.0	56.2 ± 4.9	+10.40	(+8.44, +12.36)	≥10.0°	11 (73.3%)
	FRG	46.5 ± 4.5	53.0 ± 5.1	+6.50	(+4.32, +8.68)		8 (53.3%)
UQYBT Composite (%)	MMG	85.4 ± 5.5	93.1 ± 4.8	+7.70	(+5.60, +9.80)	≥8.0%	12 (80.0%)
	FRG	86.0 ± 6.1	92.5± 5.2	+6.50	(+4.42, +8.58)		10 (66.7%)
SF-36 PCS	MMG	40.1 ± 6.3	52.5 ± 5.5	+12.40	(+9.84, +14.96)	≥5.0 points	15 (100%)
	FRG	41.5 ± 6.8	48.8 ± 6.0	+7.30	(+4.78, +9.82)		12 (80.0%)
SF-36 MCS	MMG	45.8 ± 7.0	53.9 ± 6.2	+8.10	(+5.42, +10.78)	≥5.0 points	13 (86.7%)
	FRG	46.5 ± 6.5	51.1 ± 5.8	+4.60	(+2.36, +6.84)		9 (60.0%)
